# Supplementary material for: Fusarium Head Blight in Barley from Subtropical Southern Brazil: Associated Fusarium Species and Grain Contamination Levels of Deoxynivalenol and Nivalenol
Source: Plants (Basel). 2025 Jul 27;14(15):2327. doi: 10.3390/plants14152327 (PMC12348935; doi:10.3390/plants14152327)
Supplement: Supplementary file 1 [file plants-14-02327-s001.zip › Figure S1.pdf]

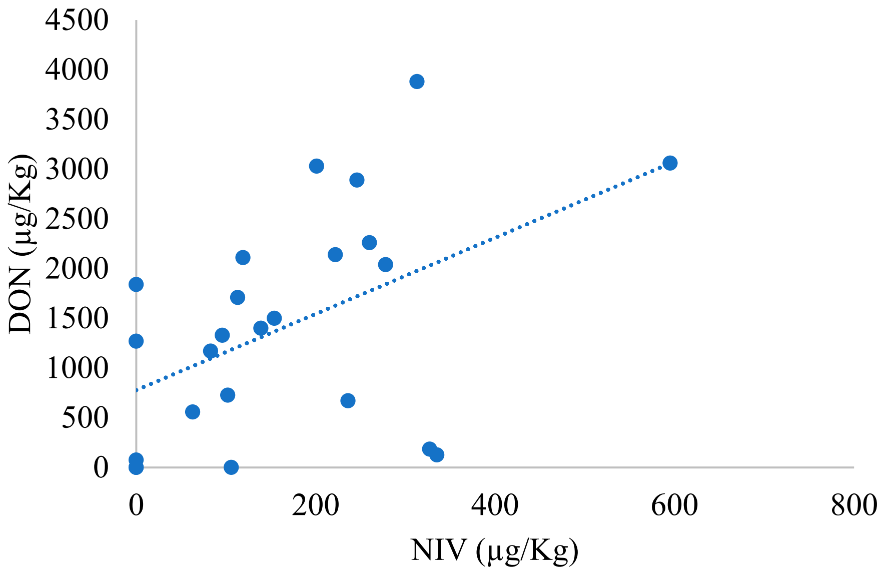

Figure S1. Distribution of deoxynivalenol (DON) and nivalenol (NIV) concentrations in barley grain samples.
