# Supplementary material for: Fusarium Head Blight in Barley from Subtropical Southern Brazil: Associated Fusarium Species and Grain Contamination Levels of Deoxynivalenol and Nivalenol
Source: Plants (Basel). 2025 Jul 27;14(15):2327. doi: 10.3390/plants14152327 (PMC12348935; doi:10.3390/plants14152327)
Supplement: Supplementary file 1 [file plants-14-02327-s001.zip › Table S1.pdf]

Table S1. Primers and PCR program used for amplification of the tef and rpb2 genes.

Gene	Primer	Direção	Sequência (5'-3')	Referência	Desnat. Inicial	Desnaturação	Anelamento	Extensão	Ext. Final	N° de ciclos
tef1	EF-1	Forward	ATGGGTAAGGARGACAAGAC	O'Donnell et al. (1998b)	94°C por 5 min	94°C por 45s	52°C por 30s	72°C por 90s	72°C por 6 min	30 ciclos
	EF-2	Reverse	GGARGTACCAGTSATCATG	O'Donnell et al. (1998b)						
rpb2	RPB2-5f2	Forward	GGGGWGAYCAGAAGAAGGC	Reeb et al. (2004)	94°C por 5 min	94°C por 45s	60°C por 45s	72°C por 1min	72°C por 8 min	5 ciclos
	fRPB2-7cr	Reverse	CCCATRGCTTGYTTRCCCAT	Liu et al. (1999)		94°C por 45s	58°C por 45s	72°C por 1min		5 ciclos
						94°C por 45s	54°C por 45s	72°C por 1min		30 ciclos
