# Supplementary material for: Fusarium Head Blight in Barley from Subtropical Southern Brazil: Associated Fusarium Species and Grain Contamination Levels of Deoxynivalenol and Nivalenol
Source: Plants (Basel). 2025 Jul 27;14(15):2327. doi: 10.3390/plants14152327 (PMC12348935; doi:10.3390/plants14152327)
Supplement: Supplementary file 1 [file plants-14-02327-s001.zip › Table S2.pdf]

Table S2. Isolates included in the phylogenetic study and their respective accession numbers in GenBank.

***Fusarium* species**	**Strain**	**GenBank accession number**	
		tef1	***rpb2***
Fusarium meridionale	LIPP 02	PQ863746	PQ863762
Fusarium graminearum	LIPP 03	PQ882731	PQ882706
Fusarium gerlachii	LIPP 04	PQ863754	PQ869613
Fusarium gerlachii	LIPP 05	PQ863755	PQ869614
Fusarium meridionale	LIPP 06	PQ863747	-
Fusarium gerlachii	LIPP 07	PQ863756	PQ869615
Fusarium graminearum	LIPP 09	PQ882732	PQ882707
Fusarium gerlachii	LIPP 10	PQ863757	-
Fusarium avenaceum	LIPP 11	PQ882770	PQ855611
Fusarium graminearum	LIPP 13	PQ882736	-
Fusarium graminearum	LIPP 14	PQ882737	PQ882711
Fusarium meridionale	LIPP 15	PQ863748	-
Fusarium graminearum	LIPP 16	PQ882738	-
Fusarium graminearum	LIPP 17	PQ882739	PQ882712
Fusarium gerlachii	LIPP 18	PQ863758	PQ869616
Fusarium graminearum	LIPP 19	PQ882740	-
Fusarium graminearum	LIPP 20	PQ882741	PQ882713

Fusarium graminearum	LIPP 25	PQ882742	PQ882714
Fusarium graminearum	LIPP 26	PQ882743	PQ882715
Fusarium graminearum	LIPP 27	PQ882744	PQ882716
Fusarium meridionale	LIPP 33	PQ863749	PQ863763
Fusarium graminearum	LIPP 34	PQ882745	-
Fusarium graminearum	LIPP 35	PQ882746	PQ882717
Fusarium graminearum	LIPP 37	PQ882747	-
Fusarium graminearum	LIPP 38	PQ882748	PQ882718
Fusarium graminearum	LIPP 39	PQ882749	-
Fusarium graminearum	LIPP 40	PQ882750	PQ882719
Fusarium graminearum	LIPP 41	PQ882751	-
Fusarium graminearum	LIPP 44	PQ882752	PQ882720
Fusarium graminearum	LIPP 46	PQ882753	PQ882721
Fusarium graminearum	LIPP 47	PQ882754	PQ882722
Fusarium asiaticum	LIPP 48	PQ855606	PQ855608
Fusarium graminearum	LIPP 49	PQ882755	PQ882723
Fusarium graminearum	LIPP 52	PQ882756	PQ882724
Fusarium graminearum	LIPP 53	-	PQ882725
Fusarium graminearum	LIPP 54	-	PQ882726
Fusarium meridionale	LIPP 55	PQ863750	PQ863764

Fusarium graminearum	LIPP 57	PQ882757	PQ882727
Fusarium avenaceum	LIPP 60	PQ882771	PQ855612
Fusarium avenaceum	LIPP 62	PQ882772	PQ855613
Fusarium asiaticum	LIPP 70	PQ855607	PQ855609
Fusarium avenaceum	LIPP 72	PQ882773	PQ855614
Fusarium cortaderiae	LIPP 75	PQ863743	-
Fusarium graminearum	LIPP 76	PQ882758	-
Fusarium gerlachii	LIPP 77	PQ863759	PQ869617
Fusarium poae	LIPP 78	PQ855619	PQ855615
Fusarium poae	LIPP 79	PQ855620	PQ855616
Fusarium gerlachii	LIPP 80	PQ863760	PQ869618
Fusarium graminearum	LIPP 81	PQ882759	-
Fusarium poae	LIPP 82	PQ855621	PQ855617
Fusarium meridionale	LIPP 83	PQ863751	PQ863765
Fusarium meridionale	LIPP 84	PQ863752	PQ863766
Fusarium graminearum	LIPP 86	PQ882760	-
Fusarium cortaderiae	LIPP 88	PQ863744	PQ882768
Fusarium graminearum	LIPP 89	PQ882761	PQ882728
Fusarium meridionale	LIPP 90	PQ863753	PQ863767
Fusarium poae	LIPP 91	-	PQ855618

Fusarium graminearum	LIPP 92	PQ882762	-
Fusarium cortaderiae	LIPP 93	PQ863745	-
Fusarium graminearum	LIPP 94	PQ882763	-
Fusarium graminearum	LIPP 95	PQ882764	PQ882729
Fusarium gerlachii	LIPP 96	PQ863761	PQ869619
Fusarium graminearum	LIPP 97	PQ882765	-
Fusarium graminearum	LIPP 98	PQ882766	-
Fusarium graminearum	LIPP 99	PQ882767	PQ882730
Fusarium graminearum	LIPP 100	PQ882733	PQ882708
Fusarium avenaceum	LIPP 101	PQ882769	PQ855610
Fusarium graminearum	LIPP 102	PQ882734	PQ882709
Fusarium graminearum	LIPP 103	PQ882735	PQ882710
